# The efficacy of transcranial alternating current stimulation for treating post-stroke depression: Study Protocol Clinical Trial (SPIRIT Compliant)

**DOI:** 10.1097/MD.0000000000019671

**Published:** 2020-04-17

**Authors:** Hongxing Wang, Wenrui Zhang, Wenfeng Zhao, Kun Wang, Zu Wang, Li Wang, Mao Peng, Qing Xue, Haixia Leng, Weijun Ding, Yuan Liu, Ning Li, Kai Dong, Qian Zhang, Xiaoqin Huang, Yunyan Xie, Changbiao Chu, Sufang Xue, Liyuan Huang, Hui Yao, Jianping Ding, Shuqin Zhan, Baoquan Min, Chunqiu Fan, Aihong Zhou, Zhichao Sun, Lu Yin, Qingfeng Ma, Andrius Baskys, Ricardo E. Jorge, Haiqing Song

**Affiliations:** a Department of Neurology, Xuanwu Hospital, Capital Medical University; b Beijing Key Laboratory of Neuromodulation; c Center of Epilepsy, Beijing Institute for Brain Disorders, Capital Medical University; d Department of Neurology, Beijing Puren Hospital; e Medical Research & Biometrics Centre, National Centre for Cardiovascular Diseases Cardiovascular; f Andrius Baskys, Graduate College of Biomedical Sciences, Western University of Health Sciences, Pomona, CA; g Department of Psychiatry and Behavioral Sciences, Baylor College of Medicine, Houston, TX.

**Keywords:** post-stroke depression, randomized controlled trial, study protocol, transcranial alternating current stimulation

## Abstract

**Background:**

The treatment of post-stroke depression (PSD) with anti-depressant drugs is partly practical. Transcranial alternating current stimulation (tACS) offers the potential for a novel treatment modality for adult patients with PSD. In this study, we will assess the efficacy and safety of tACS for treating PSD and explore its effect on gamma and beta-oscillations involving in emotional regulation.

**Methods:**

The prospective study is an 8-week, double-blind, randomized, placebo-controlled trial. Seventy eligible participants with mild to moderate PSD aged between 18 years and 70 years will be recruited and randomly assigned to either active tACS intervention group or sham group. Daily 40-minute, 77.5-Hz, 15-mA sessions of active or sham tACS targeting the forehead and both mastoid areas on weekdays for 4 consecutive weeks (week 4), and an additional 4-week observational period (week 8) will be followed up. The primary outcome is the proportion of participants having an improvement at week 8 according to the Hamilton Depression Rating Scale 17-Item (HAMD-17) score, including the proportion of participants having a decrease of ≥ 50% in HAMD-17 score or clinical recovery (HAMD-17 score ≤ 7). Secondary outcomes include neurological function, independence level, activities of daily living, disease severity, anxiety, and cognitive function. The exploratory outcomes are gamma and beta-oscillations assessed at baseline, week 4, and week 8. Data will be analyzed by logistical regression analyses and mixed-effects models.

**Discussion:**

The study will be the first randomized controlled trial to evaluate the efficacy and safety of tACS at a 77.5-Hz frequency and 15-mA current in reducing depressive severity in patients with PSD. The results of the study will present a base for future studies on the tACS in PSD and its possible mechanism.

Trial registration number: NCT03903068, pre-results.

## 1 Introduction

Post-stroke depression (PSD),^[1]^ as a clinical syndrome following brain injury, is the most common complication of stroke and is characterized by prominently depressed mood, markedly diminished interest or pleasure, overwhelmed feeling of isolation and despair.^[1–7]^ The prevalence of PSD is high with approximately one-third of stroke survivors at any one time,^[8]^ spanning from 39% to 52% within the first 5 years.^[9]^ PSD can negatively affect patients, including poor functional outcomes^[3,8]^ and increased mortality.^[3,5,10]^ Some therapeutic options have shown to be effective for PSD, including pharmacological,^[11]^ non-pharmacological interventions especially for late-life depression,^[12]^ and combination therapies.^[11,13–17]^ The most available agents are antidepressants, mainly selective serotonin reuptake inhibitors (SSRIs) and serotonin and norepinephrine reuptake inhibitors (SNRIs).^[14]^ However, those antidepressant agents not only show unwanted side effects, including nausea, diarrhea, fatigue, and dizziness,^[3]^ but also produce a high chance of hemorrhagic complications and stroke.^[18,19]^ Therefore, in addition to antidepressants treating PSD, non-pharmacological interventions have been proposed to treat PSD,^[4,13]^ including transcranial magnetic stimulation (TMS). Since the Food and Drug Administration approved TMS for the treatment of major depressive disorder (MDD) in 2009,^[20]^ the increasing interest in the effect of new non-pharmaceutical therapy for depression has been reported.^[21]^ However, the TMS procedure in different trials not only has paradoxical consequences,^[22]^ but also causes a potential of seizure,^[23]^ as well as has relatively high costs.^[21]^

Transcranial alternating current stimulation (tACS) is a kind of cranial electrotherapy stimulation (CES). Studies revealed that it is a noninvasive, portable, safe,^[24]^ easy to manage, and well-tolerated^[25]^ CES of delivering brain stimulation by applying a low intensity electrical current to the scalp to modulate cortical excitability and spontaneous brain activity.^[26–29]^ Compared with transcranial direct current stimulation (tDCS), tACS has fewer adverse events, and only mild and temporary adverse reactions were reported.^[30]^ Accordingly, it has obtained increasing attention on the effect of regulating brain activity in different participants,^[31,32]^ and has been suggested to treat anxiety, depression, and insomnia.^[23]^ However, there is a lack of robust results available to support the effect of tACS treating patients with post-stroke depression.^[33]^

Regarding the mechanism of tACS modulating brain activity, the published papers showed that tACS may target alpha-, gamma-, and theta- oscillations to play important roles in manipulating brain function. ^[34–37]^ However, studies exploring the application of tACS practices for oscillations-based outcomes in PSD are scarce. The recent report indicated that tACS can treat depressive symptoms by modulating alpha-oscillations in MDD patients.^[37]^ However, to our knowledge, no published reports are assessing the oscillations-based mechanisms underlying the treatment effects of tACS in patients with PSD.

Our preclinical experiment on the effect of tACS at a frequency of 77.5-Hz and 15-mA current treating in depression have shown that tACS has a therapeutic effect via stimulating the forehead and both mastoid areas.^[38]^ Therefore, we hypothesize that tACS at a frequency of 77.5-Hz and 15-mA current may treat patients with PSD. And the proposed study is to explore the efficacy of the tACS at a frequency of 77.5-Hz and 15-mA current in the treatment of depression in PSD participants. The study will provide an alternative approach to treat individuals with PSD.

## 2 Methods

### 2.1 Study design

**Figure 1:**
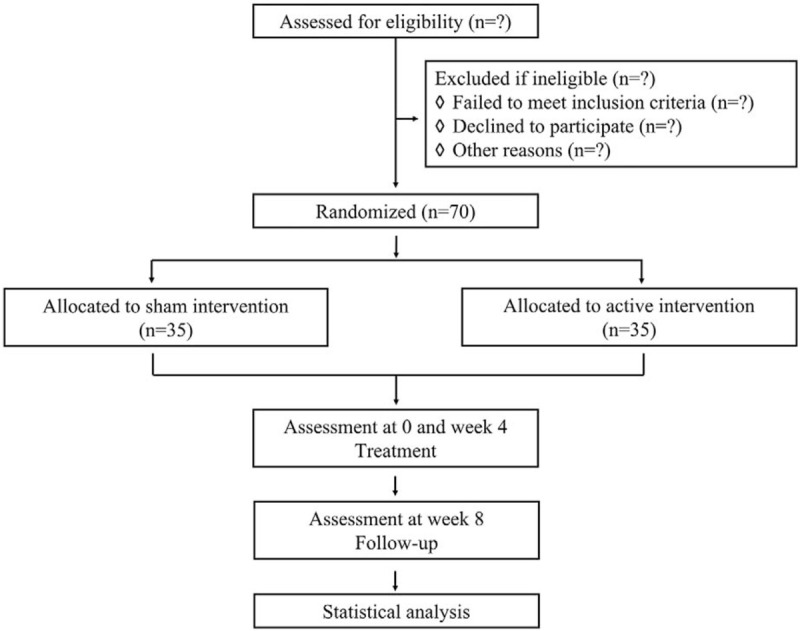
Flowchart of the study design.

This is an 8-week, randomized controlled trial (RCT) aimed to evaluate the efficacy of tACS in treating PSD. Seventy participants with PSD will be recruited from the Department of Neurology, Xuanwu Hospital of Capital Medical University, Beijing, China. After obtaining written informed consent from participants, potential participants will be screened for their eligibility. If eligible, they will be randomized to either the active or sham intervention group with a ratio of 1:1. Participants will undergo a 4-week intervention period (week 4), and another 4-week observational follow up (week 8). Outcomes will be evaluated for all participants at 3 time points: at baseline, at week 4, and week 8. All procedures of this trial will follow the strictly Consolidated Standards of Reporting Trials (CONSORT) guidelines (see Fig. 1) and Standard Protocol Items: Recommendations for Interventional Trials (SPIRIT) Checklist.

### 2.2 Recruitment

Trained investigators will explain the study's objectives, procedures, and possible side effects of participation in detail for each potential participant, who has the right to ask any questions and obtain corresponding answers before he or she makes a decision. After a participant agrees to enroll, a written informed consent form should be obtained, and another copy of the content form will be provided for the participant. Each potential participant will be assessed for his or her eligibility by a trial investigator. If the potential participant is eligible, they will be randomized into tACS or sham treatment. An outline of the procedures is presented in Figure 1. The trial will recruit participants beginning at March 15, 2020, will complete at August 30, 2020.

### 2.3 Inclusion and exclusion criteria

A diagnosis of PSD is based on the “Depressive disorder due to another medical condition” of Diagnostic and Statistical Manual of Mental Disorders, Fifth Edition (DSM-V).^[39]^ Inclusion criteria are as follows: aged 18 to 70 years of either sex, right-handed, more than 6 months after the onset of stroke; satisfying with the criteria of major depressive disorder with single episode,^[39]^ the duration of depressive disorder persists for more than two weeks; having the Hamilton Depression Rating Scale 17-Item (HAMD-17) scores higher than 17 at baseline;^[40]^ absence of psychiatric disorder or family history of psychosis before stroke; has never taken antidepressants before enrollment; having the level of audiovisual for examinations required for the study, and providing signed informed consent.

Exclusion criteria include: patients with life expectancy < 6 months; severe or unstable organic diseases, acute brain injury and infection; the impaired skin integrity at the electrode placement site or skin allergic to electrode gel or adhesive; active current suicidal intent or plan as shown by a score of ≥3 on the suicide item of HAMD-17; current participation in any other clinical trial; prior exposure to all kinds of neuromodulation treatments (including electroconvulsive therapy, TMS, tDCS, etc); prior exposure to any implanted device in body (including a cochlear implant, cardiac pacemaker, an implanted device or metal in the brain); a history of brain organic diseases (including seizures, hydrocephalus, and brain tumors), and any situations the investigators believe that they are not suitable for this study.

### 2.4 Withdrawal and discontinuation

Participants can freely withdraw the trial at any time. Criteria for the study termination include: severe adverse responses; other diseases for which treatment may affect the assessment of the tACS; absence of two consecutive tACS sessions; nonattendance at weeks 4 and 8; and withdrawal of consent.

### 2.5 Intervention

Participants will be allotted to receive active or sham tACS intervention (Nexalin Technology, Inc., Houston, TX) under the same instructions during all sessions for 4 weeks in addition to their routine therapies (including antiplatelet, lipid-lowering, antihypertensive, or antidiabetic therapy). Two trained nurses will conduct the intervention and instruct participants to receive tACS. Participants will be encouraged to turn off their smartphones, drink water, and relax, even fall asleep. Communication with the trained nurses will be restricted.

Three electrodes will be positioned to the scalp: one 4.45 cm × 9.53 cm electrode will be placed over the forehead (Fpz, Fp1, and Fp2, in the 10/20 international placement system) and two 3.18 cm × 3.81 cm over the mastoid areas. Each participant will receive 20 sessions of intervention in 4 consecutive weeks at the fixed time frame once daily from Monday through Friday, with weekends off. Each session lasts 40 minutes with the default current level of 15-mA and a patented frequency of 77.5-Hz in the active group and no stimulation in the sham group. Then, patients will have another 4 weeks of follow-up.

Participants will be guided to refrain from any antidepressants for treating PSD during the study, and can willingly withdraw from this study at any time and choose any other medical treatments, such as antidepressants, when their depressive symptoms worsen.

### 2.6 Outcomes and measures

#### 2.6.1 Primary outcomes

The primary outcome is the proportion of participants having an improvement at week 8, which includes the response per HAMD-17 defined as a ≥ 50% reduction from the baseline^[41]^ or clinical recovery (score ≤ 7).^[21]^

#### 2.6.2 Secondary outcomes

The proportions of participants achieve an improvement in neurological function at weeks 4 and 8 during the trial. The improvement will be decided by a reduction of ≥ 50% or the total score of 0–1 in the National Institute of Health Stroke Scale (NIHSS) score (ranging from 0 to 42, higher scores indicate a more severe neurological deficit).^[42]^

The proportions of participants achieve an improvement in independence at weeks 4 and 8, as measured by a modified Rankin Scale (mRS) over the trial (scores on this scale range from 0 to 6, with higher scores indicating more significant disability),^[43]^ and the improvement is defined as 0, 1, and 2 in mRS.

The proportions of participants with a Barthel Index (BI) score of ≥90 at weeks 4 and 8 during the study. The BI is used to assess the activities of daily living (ranging from 0 to 100, higher scores indicate increased independence).^[44]^

The proportions of participants have improvements in the Clinical Global Impression scale (CGI). The CGI includes the severity scale (CGI-S) and the improvement scale (CGI-I), both are 7-point rated scales, with lower scores indicating less illness and more improvement, respectively.^[45]^

The changes of participants on anxiety symptoms at weeks 4 and 8 assessed by the Hamilton Anxiety Rating Scale (HAMA), and it ranges from 0 to 56, and higher scores indicate more anxiety.^[46]^

The changes of participants on cognitive function at weeks 4 and 8, as evaluated by the Mini-Mental State Examination (MMSE) (range from 0 to 30, higher scores indicate impaired cognition)^[47]^ and the Montreal Cognitive Assessment (MoCA) (ranging from 0 to 30, and higher scores indicate impaired cognition in different cognitive domains).^[48]^

The changes of beta-and gamma-oscillations at weeks 4 and 8, as assessed via resting-state high-density electroencephalogram (rsHEEG) by utilizing a 128 channel electroencephalogram (EEG) system (Geodesic EEG system 400, Electrical Geodesics, Inc., OR) at baseline, week 4, and week 8. Gamma and beta-oscillations induced by tACS correlate with aspects of executive function and emotional regulation.^[49,50]^

The variations of cognitive status at weeks 4 and 8, as measured by the repeatable battery for the assessment of neuropsychological status (RBANS), which includes different domains of attention, immediate and delayed memory, language, and visuospatial/constructional. And the Chinese version of RBANS has been certified as a reliable tool for cognitive function assessment with reasonable reliability and validity.^[51]^ The higher scores indicate the better in the neurocognitive state.

### 2.7 Adverse effects

Adverse events will be recorded on Case Report Forms (CRF) though the overall trial at weeks 4 and 8, according to vital signs, the treatment-emergent symptom scale (TESS) and EEG.

The proportions of participants who have symptoms in the TESS will be analyzed at weeks 4 and 8. TESS is composed of behavioral toxicity, laboratory abnormalities, nervous system, automatic nervous system, cardiovascular system and six other aspects (ranging from 0 to 4, the higher score, the more serious the adverse reactions).

The proportions of participants have an epileptic seizure based on EEG activity and clinical manifestations at weeks 4 and 8.^[52]^ At the request of the local ethics committee, EEG recordings will be monitored for each participant in the neurophysiology lab of the Department of Neurology of Xuanwu Hospital, Capital Medical University according to the electrode locations of the international 10/20 system for EEG recording at baseline and weeks 4 and 8. All participants will be recorded by EEG equipment for more than 30 minutes,^[29]^ and whether the participant will have an epileptic seizure will be assessed by EEG professional technicians and investigators.

### 2.8 Study visits and procedures

The visit schedule for all assessments (baseline, at weeks 4 and 8) is presented in Table 1. Participants will be evaluated by the two independent raters, who are blind to group assignment, at each observational timepoints. Baseline assessments, demographic characteristics, and medical history will be gathered at the first visit only. All concurrent medications will be recorded during the 4-week intervention period (i.e., baseline to week 4) and the another 4-week follow-up period (i.e., week 4 to week 8), including the name, dosage, and course of these medications. Primary and secondary outcomes and safety will be collected at weeks 4 and 8.

**Table 1:**
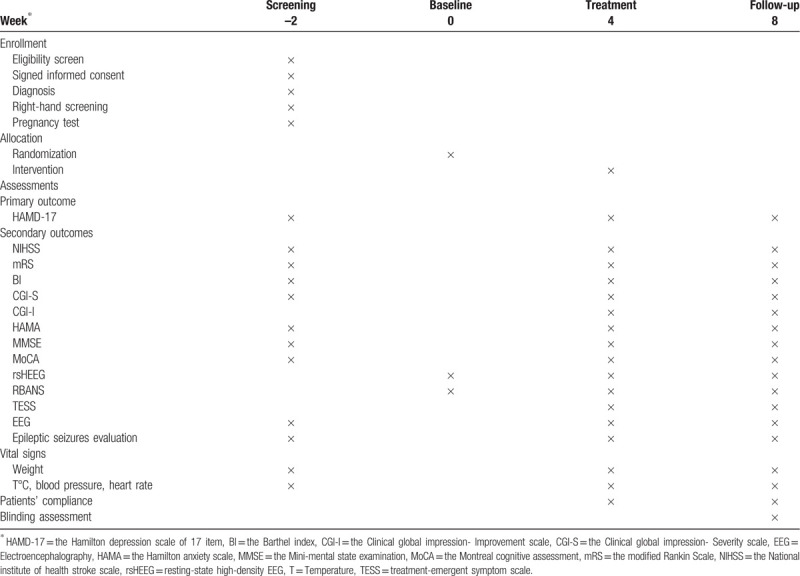
Schedule of the enrollment, interventions, and assessments in the trial.

### 2.9 Randomization and blinding

An independent statistician, who is not be involved with the enrolment nor the assessment of the study participants, will establish and keep the concealed randomization schedule using a computer-generated random number sequence. The randomization sequence will be computed with the blocks of 4. Participants will be randomized into 2 groups with a ratio of 1:1, and the active and sham groups will be coded A and B, respectively. The code will be set in an opaque envelope and subsequently sealed. 10 tACS devices (5 sham and 5 active) have the same size, color, appearance, weight, and odor, and are coded as A or B. If one participant is allotted to A, only devices coded as A will be used through the course of the intervention, similarly if B, only B devices can be utilized. Two trained nurses of tACS devices will know the tACS devices’ status, but not involved in the assessment of participants and will keep blindness to participants and other study staff about tACS devices. Persons involving in the study, including the investigators, raters, EEG technicians, participants, trial monitors, and statistician, will be blinded to treatment assignment during double-blinded treatment until the end of follow-up.

### 2.10 The integrity of blinding

The integrity of trial-group blinding will be evaluated by asking participants and the blinded raters separately to guess which intervention each participant receives. Blinding will have to be broken in case of an emergency.

### 2.11 Data monitoring

The trial will be performed according to the International Conference on Harmonization Guidelines for Good Clinical Practice to assure the rigor of the study. Data will be collected in the paper-based CRFs at baseline, weeks 4 and 8 (Table 1). An independent party approved by the local ethical committees will be responsible for data management. All staff is asked to join in a series of training sessions that will cover communicating with participants on trial, handling assessments, instructing participants to relax and drink water during the tACS intervention, collecting variables, and completing CRF. Data will be entered into the EpiData system by two independent investigators, and double-entry verification will be conducted. Any inconsistent entries will be re-checked with original CRF or investigators’ source data if necessary. Typos will be revised until two entered databases are the same. Auditing will be performed for the trial via frequent visits by two neutral trial monitors who have been appointed by the local ethics committee. The independent statistician will have access to the final trial dataset, including intent-to-treat dataset (i.e. all enrolled participants as belonging to the group they will be randomized into) and per-protocol dataset (i.e. those participants who will complete the trial originally allocated).

### 2.12 Compliance and retention

For maximizing compliance and retention over the trial, the information on the entire trial (including the study schedule, requirements, potential risks, and benefits) will be fully explained to all interested participants. The signed informed consent form will be obtained before the initiation of tACS intervention. All patients will receive a call for their exact appointments before their assessments at weeks 4 and 8. Additionally, we will provide patients ongoing treatment regimens for their brain vascular diseases, free transportation allowance, and free relative examination. After the trial, we will continue to provide patients treatments for their vascular diseases in our outpatient clinic.

### 2.13 Sample size calculation

The sample size is estimated by assuming a 50% improvement rate at the end of 8 weeks in the active group and a 15% in the sham group, which were based on our pilot trial of the effect of tACS in adults with major depression disorder.^[38]^ Thus, each group will be recommended to have a minimum sample size of 26 with a power to 80% and a 2-tailed α level of 5%.^[53]^ Considering the 20% attrition rate, 32 evaluable participants will be required for each group. And the total sample size will be 70.

### 2.14 Statistical analysis

All data from the study will be analyzed by an independent biostatistician using SAS statistical software, version 9.4 (SAS Institute Inc) and will be considered statistically significant at a *P* value < .05, and all tests are two-sided. Outcomes are based on the intention-to-treat analysis. Missing data will be monitored using the multiple imputation method. Continuous variables will be summarized as mean and standard deviation (SD). Categorical variables will be described as frequency and percentage. Between-group comparisons will be tested by the Mann-Whitney *U* test for continuous variables and the Chi-square test for categorical variables.

The primary outcome will compare the proportion of participants achieving an improvement of depression per HAMD-17 between the 2 groups after 8 weeks of the trial. Secondary outcome analyses will be conducted based on standard statistical principles for comparison of parametric or non-parametric distributions as appropriate. Logistical regression analyses will be utilized to analyze the primary and secondary variables between groups, such as HAMD-17 total score, NIHSS score, mRS score, BI score, CGI-I score, CGI-S score, and epileptic seizure. The secondary outcomes on changes of HAMA, MMSE, MoCA, RBANS, and beta-and gamma-oscillations will be analyzed using mixed-effects models, controlling for time of measurement.

### 2.15 Ethics

The study has received full ethical approval from the Ethics Committee of Xuanwu Hospital, Capital Medical University, Beijing, China (LYS[2018]-092) on September 5, 2018, then amended (LYS[2018]-092-Amendment 1) on May 15, 2019. And the ethics committee's phone, email, and address as follows: 0086-10-83919270, xwkyethics@163.com, and No. 45, Changchun Street, Xicheng District, Beijing 100053, China. The study is registered in the ClinicalTrials.gov (NCT03903068). Participants will provide written informed consent to enroll before the trial and voluntarily withdraw consent or cease to enroll at any time for any reason. The results will be showed at international conferences and published in peer-reviewed journals.

## 3 Discussion

Depression is a common complication after stroke and is associated with poor functional outcome and high mortality.^[1,2,4]^ The relation between depression and stroke has been established, and stroke has been shown to elevate the risk of PSD, and conversely, depression is an independent risk factor for stroke.^[4,5,54]^ The most commonly applied antidepressants are SSRI and SNRIs, although having side effects including increased risk of hemorrhagic complications. Because antidepressant agents have the potential to cause a series of adverse effects and are related to poor patient compliance, it is crucial to explore other effective treatments that may have fewer side effects in the management of PSD.

In this trial, we will explore whether the tACS can lessen depression among subjects who have suffered a stroke. Although the efficiency of tACS in improving depression has been proposed as promising evidence,^[49,55–62]^ no evidence with modern rigor methodology has been provided.^[33]^ The therapeutic effect of tACS intervening brain has not been fully understood, it may involve in stimulation sites and neurotransmitter mechanisms.^[63–65]^ This study will be performed as an RCT to provide evidence for the clinical efficacy of the tACS in the treatment of PSD. The outcome of the study will offer evidence-based data regarding whether the tACS is beneficial for participants with PSD. Additionally, brain activities including beta- and gamma-oscillations of PSD participants will be explored in our trial for the possible mechanism of the tACS treating PSD. The results of the trial will be reported at international meetings and published in peer-reviewed journals. Notably, the success of this study will provide a large-scale clinical study to further consolidate the evidence for the use of the tACS in PSD patients.

This trial has some limitations that require consideration. For example, the study is being undertaken in a single center; therefore, it may not necessarily be possible to predict the results from the trial to other regions or ethnic groups. Second, the intervention duration is short.

In conclusion, this prospective trial will present that the tACS can effectively and safely treat PSD, consequently providing a new direction for treating PSD with minimal side effects.

## Acknowledgments

The authors are grateful for Nexalin Technology, Inc., Houston, TX, USA. Freely provided medical devices.

## Author contributions

HW and HS drafted the study protocol. HW is responsible for the ethics application and reporting. HW and HS are responsible for recruitment and data collection. HW will take a lead role in preparing publications on the clinical outcomes of the study. HW, WZ, KW, and HS will contribute to the preparation of publications and are providing supervision throughout the study. LY will take on a lead role for statistical analysis. HW drafted the final version of this manuscript, while all authors critically reviewed and approved the final version.
